# Checks and balances occur not only in government, but also in biology

**DOI:** 10.18632/oncotarget.25876

**Published:** 2018-09-04

**Authors:** John T. Isaacs

**Affiliations:** John T. Isaacs: Department of Oncology, Prostate Cancer Program, The Sidney Kimmel Comprehensive Cancer Center at Johns Hopkins, Baltimore, MD, USA; The Brady Urologic Institute, Department of Urology, The Johns Hopkins University School of Medicine, Baltimore, MD, USA

**Keywords:** GHRH, super agonists, IGF-1, cancer

Throughout evolution, endocrine physiology has utilized the development of competitive positive and negative receptor mediated feedback signaling to produce checks and balances for the appropriate regulation of host growth and reproduction based upon environment inputs. The importance of such checks and balances was acknowledged by the awarding of the Nobel Prize in Physiology and Medicine in 1977 to Roger Guillemin and Andrew V. Schally for their discoveries concerning the agonist peptide releasing hormones as the main link between the brain and the pituitary as the central regulator control of reproductive functions in mammals [1]. Building upon these original discoveries, we now know that in the male, when ligand binding to the androgen receptor is low in neurons in the hypothalamus, they secrete a peptide luteinizing hormone (LH) releasing hormone LHRH into the pituitary microcirculation where it binds to its cognate receptor stimulating pituitary production and secretion of LH hormone into the systemic circulation. Once in the blood, LH binds to its receptor on the Leydig cells in the testes stimulating their secretion of testosterone (T) into the blood. To prevent serum T from continuously increasing via such a LHRH dependent positive feedforward loop in the male, there has developed a counteractive T dose dependent negative feedback loop producing a check and balance to control LHRH secretion. This negative feedback is activated when serum androgen reaches its physiologic normal level allowing sufficient ligand binding of the androgen receptor in the hypothalamus to cause down regulation of its LHRH release, decreasing pituitary LH release and thus decreasing Leydig cells secretion of T.

Based upon these understandings, LHRH peptide “super” agonist analogs have been synthesized incorporating modified amino acids to stabilize them from the usual rapid proteolytic degradation of the natural agonist [1]. An unexpected discovery about such a check and balance system for the regulation of serum T is the “paradoxical” inhibitory effects following a continuous exogenous administration of such LHRH super-agonists. The mechanism for such agonist inhibition is related to the fact that secretion of LHRH is pulsatile. Thus, for a physiologic stimulation of secretion of LH, an intermittent LHRH release, every 30-120 minutes is necessary [2]. A sustained stimulation of the pituitary by repeated injections of LHRH agonists or periodic administration of microcapsules (depot preparations) of LHRH agonists induces suppression of the pituitary-testis axis through a reduction in the number of pituitary receptors for LHRH, decrease in the expression of mRNA for LHRH receptor, rapid homologous desensitization of LHRH receptors and inhibition of blood levels of LH, and serum T [3]. Thus, this process of downregulation of pituitary LHRH receptors induced by super LHRH-agonists overcomes the normal checks and balance of the hypothalamic-pituitary-testis axis to create a state of reversible medical castration which has become standard of care for androgen deprivation therapy for metastatic prostate cancer [1].

Such a paradoxical overcoming of normal endocrine checks and balances by a pituitary super-releasing hormone agonist is not limited to LHRH analogs. For example, Tengjiao Cui and Andrew V. Schally report in this issue of ONCOTARGET that treatment with GHRH super-agonists have anti-cancer efficacy against human cancer xenografts via their ability to inhibit the synthesis and release of Insulin-like Growth Factor 1(IGF-l) by normal liver tissue and in tumors [4]. These are exciting results since IGF-1 stimulates cancer cell proliferation, inhibits cancer cell apoptosis and facilitate metastasis by promoting cell migration and invasion [5]. Most circulating IGF-1 is produced by the liver, following the stimulation with pituitary growth hormone [GH] [6]. High levels of circulating IGF-1 have been linked to an increased risk of neoplasia and poor prognosis in many clinical studies [5]. While previous studies have documented the secretion of IGF-l in physiological, clinical and pathological conditions [7], the discovery by Cui and Schally of a neurohumoral component in these situations is both novel and significant in that it opens a variety of novel approaches using GHRH super-agonists for solid malignancies.
